# Association between Air Pollutants and Asthma Emergency Room Visits and Hospital Admissions in Time Series Studies: A Systematic Review and Meta-Analysis

**DOI:** 10.1371/journal.pone.0138146

**Published:** 2015-09-18

**Authors:** Xue-yan Zheng, Hong Ding, Li-na Jiang, Shao-wei Chen, Jin-ping Zheng, Min Qiu, Ying-xue Zhou, Qing Chen, Wei-jie Guan

**Affiliations:** 1 Department of Epidemiology, School of Public Health and Tropical Medicine, Southern Medical University, Guangdong, China; 2 State Key Laboratory of Respiratory Disease, National Clinical Research Center for Respiratory Disease, Guangzhou Institute of Respiratory Disease, The First Affiliated Hospital of Guangzhou Medical University, Guangzhou Medical University, Guangzhou, China; 3 Department of Biostatistics, School of Public Health and Tropical Medicine, Southern Medical University, Guangdong, China; Hasselt University, BELGIUM

## Abstract

**Background:**

Air pollution constitutes a significant stimulus of asthma exacerbations; however, the impacts of exposure to major air pollutants on asthma-related hospital admissions and emergency room visits (ERVs) have not been fully determined.

**Objective:**

We sought to quantify the associations between short-term exposure to air pollutants [ozone (O_3_), carbon monoxide (CO), nitrogen dioxide (NO_2_), sulfur dioxide (SO_2_), and particulate matter ≤10μm (PM_10_) and PM_2.5_] and the asthma-related emergency room visits (ERV) and hospitalizations.

**Methods:**

Systematic computerized searches without language limitation were performed. Pooled relative risks (RRs) and 95% confidence intervals (95%CIs) were estimated using the random-effect models. Sensitivity analyses and subgroup analyses were also performed.

**Results:**

After screening of 246 studies, 87 were included in our analyses. Air pollutants were associated with significantly increased risks of asthma ERVs and hospitalizations [O_3_: RR(95%CI), 1.009 (1.006, 1.011); I^2^ = 87.8%, population-attributable fraction (PAF) (95%CI): 0.8 (0.6, 1.1); CO: RR(95%CI), 1.045 (1.029, 1.061); I^2^ = 85.7%, PAF (95%CI): 4.3 (2.8, 5.7); NO_2_: RR(95%CI), 1.018 (1.014, 1.022); I^2^ = 87.6%, PAF (95%CI): 1.8 (1.4, 2.2); SO_2_: RR(95%CI), 1.011 (1.007, 1.015); I^2^ = 77.1%, PAF (95%CI): 1.1 (0.7, 1.5); PM_10_: RR(95%CI), 1.010 (1.008, 1.013); I^2^ = 69.1%, PAF (95%CI): 1.1 (0.8, 1.3); PM_2.5_: RR(95%CI), 1.023 (1.015, 1.031); I^2^ = 82.8%, PAF (95%CI): 2.3 (1.5, 3.1)]. Sensitivity analyses yielded compatible findings as compared with the overall analyses without publication bias. Stronger associations were found in hospitalized males, children and elderly patients in warm seasons with lag of 2 days or greater.

**Conclusion:**

Short-term exposures to air pollutants account for increased risks of asthma-related ERVs and hospitalizations that constitute a considerable healthcare utilization and socioeconomic burden.

## Introduction

Asthma is characterized by airway hyperresponsiveness and inflammation, the pivotal components leading to the cascades of pro-inflammatory mediator release and airflow limitation [1] that are associated with allergen exposures, air pollution, cigarette smoking and noxious particle insults [2].

The relationship between air pollution and asthma has been well-established [3–89], particularly in the countries with rapid urbanization and industrialization. Three multi-center studies conducted in Europe [14,51] and Australia [53] reported an overall insignificant association between major air pollutants and the asthma-related emergency room visits (ERVs), except for nitrogen dioxide (NO_2_) [14,53] and particulate matter with a diameter of 10 μm or less (PM_10_) [51]; whereas other multi-city studies conducted in Korea and Europe demonstrated different magnitudes of the associations between asthma exacerbation and ozone (O_3_) [5] and sulfur dioxide (SO_2_) [5,76] pollution. Moreover, exposure to environmental NO_2_ and PM_10_ has recently been associated with worsening of symptoms and lung function decline during asthma exacerbations [90–92].

Whilst the adverse impacts of air pollution on asthma exacerbations have been confirmed, the effect sizes and the extent to which any single pollutant acts as a surrogate of other pollutants are less clear. As epidemiologic evidence regarding the effects of air pollution on asthma accumulates, it is crucial to consider different concentration-response functions (CRFs, defined as the percentage change in any health outcome per unit change in concentration, to different air pollutants [93]), based on the concurrent evidence. Determination of the effect modification across studies may also be challenging because of the underlying geographic diversity, heterogeneous primary outcome indices, the differences in statistical algorithms, the complexity of multiple pollutants and other confounders [4].

Consequently, meticulous risk assessments exploring the influences of multiple air pollutants, calculated as the CRFs [93], are warranted. In view that the quantification between air pollution and asthma-related ERVs or hospitalizations has been well-established and that the majority of population is exposed to air pollution, the relative risks (RRs) and population attributable fractions (PAFs) of individual pollutants on asthma-related ERVs or hospitalizations should be taken into account. Furthermore, investigations of the effect modification may provide further insights into these associations [94]. For instance, there have been the literature reports delineating stronger pollution effects during the warm seasons, despite the culmination of pediatric asthma attacks during cold seasons [1,4,5,17,18,24,52,54,85]. Sex [5,7,37,40,50,68] and age [16,24,30,38,57] differences might also confound the asthma outcomes to air pollutant exposure.

In this study, we sought to conduct a systematic review and meta-analysis on the association between short-term exposure to air pollutants and asthma-related ERVs and hospital admissions based on time-series and case-crossover studies, thus offering rationales to improve public health and environmental protection. We further assessed the impacts of age, sex, season, hospital variance and long lag patterns (lag >2days) on these associations.

## Methods

### Eligibility criteria and literature searches

Systematic searches were conducted to identify studies focusing on short-term exposures, defined as the duration of up to 7 days to one of the air pollutants associated with asthma exacerbation. These studies involved all age intervals without language limitation. We excluded: (1) animal studies, *ex vivo* and toxicological studies, summaries, commentaries and editorials, case reports and case series; (2) duplicate publications; (3) studies evaluating long-term exposure only; (4) non-peer reviewed articles (a potential source of bias); (5) study duration of less than one year; (6) no original data. Authors were contacted by e-mail in case data were incomplete. Studies were excluded if no reply was obtained despite repeated contacts with corresponding authors.

Time-series studies (including case-crossover studies) were searched comprehensively in EMBASE, PubMed, Cochrane Central Register of Controlled Trials and EBM Reviews–Cochrane Database of Systematic Reviews, Web of Science, Ovid and Highwire up to March 2015 (no start date specified). References were checked for additional data. When the same population was used in several publications, only the largest and the most complete study (i.e. multi-cities study) was included. In addition, single-city study with different time periods from multi-cities study was also accepted. We used a combination of keywords related to the types of exposure (air pollution, CO, PM_10_, PM_2.5_, SO_2_, NO_2_ and O_3_) and the outcomes of asthma exacerbations (hospital admission and ER visit). (See ***Search Strategies*** in the online supplements for further details)

### Quality score assessment

This study complies with the preferred reporting items of PRISMA [95]. Since no validated scales of time-series and case-crossover studies were recommended by New Castle Ottawa and Cochrane risk of bias tool, we evaluated the validity based on Mustafic’s study [96]. Three components were assessed, including asthma diagnosis (0 to 1 point), air pollutant measures (0 to 1) and adjustment for confounders (0 to 3). We confirmed asthma exacerbation if coded by *International Classification of Diseases*, *American Thoracic Society*, *National Asthma Education or Prevention Program or International Classification of Primary Care* 2 (0 for no valid criteria). The frequency of measurement and missing data were considered (1 point for measurements performed daily with <25% missing data, otherwise 0 point was assigned). Regarding the potential confounders, 1 point was scored if long-term trends, seasonality and temperature were all adjusted, otherwise 0 point was assigned. Any additional adjustment for the humidity or day-of-week was added for 1 point. Any adjustment made for influenza epidemics and holidays was added for 1 point. Studies fulfilling 5 points were analyzed in sensitivity analyses.

### Study selection and data extraction

Two independent reviewers (S.C. and H.D.) screened the abstracts and titles. Full texts were reviewed to determine eligibility for inclusion. Disagreement was resolved by discussion. If consensus was not reached, another reviewer (X.Z.) was consulted to vote for final decisions.

A standardized form was used for data extraction including the main characteristics (author, year of publication, location and period, type of study, age and sex of populations, title and journal), outcome measures (general practitioner’s house calls, primary care visits, asthma-related hospitalization and ERV), the quality of measurement methods and adjustments (long-term trend, seasonality, temperature, humidity, days of the week, holidays and influenza epidemics). Data extraction was done by two independent reviewers (W.G. and L.J.) for comparisons. Disagreements were resolved by consultation with the third reviewer (X.Z.).

### Statistical analysis

As current evidence suggests a linear association between air pollutants and asthma-related ERVs and hospital admissions, the standardized effect estimates were expressed as the risk ratios (RRs) and 95% confidence intervals (95%CIs), derived from single-pollutant models reporting RRs (95%CIs) or percentage change (95%CIs), and further recalculated to reflect a 10 μg/m^3^ increase in the pollutants by assuming a linear relationship of all pollutants, except for CO (1 mg/m^3^ increase) [96]. There was a time lag (measured in days) between short-term air pollution and asthma exacerbations; however, each of the included study varied in lag selection patterns. Some authors recommended the use of the most significant estimate, irrespective of the direction. Given the lack of standardized methods of reporting, we adopted a *priori* lag selection scheme proposed by Atkinson et al [97]. If only one lag estimate for a given pollutant/outcome pair was demonstrated (either the only one was analyzed or reported), it would be included for analyses. If multiple lag estimates were reported, the selection algorithms were: 1) the most frequently used lag in all selected studies; 2) single lags, but not cumulative/distributed lags, were selected as a priority.

Random-effect model is the most conservative tool incorporating within- and between-study heterogeneity in 95%CI, and has been adopted for studies investigating different populations with anticipated significant heterogeneity which is calculated using the I^2^ test. The I^2^ values of 0 to 30, 30 to 50, and greater than 50 denoted low, moderate and high heterogeneity, respectively [98]. This algorithm is currently recommended by Cochrane collaboration (http://www.cochrane.org) despite concerns of underpowered statistics.

The prevalence of exposure to air pollution in the population was estimated to be 100%, which is imputed from the epidemiological studies reporting effect estimates [99]. Population attributable fractions (PAFs) were calculated from RRs (95%CI) in overall analyses, calculated as PAF = (RR−1)/RR.

To explore the heterogeneity in our pooled analysis, sensitivity analyses of the lag patterns and study quality were applied, based on the studies with the same and most commonly used lag pattern or the studies with 5 scores.

Subgroup analyses of the study characteristics were conducted to combine the effects for evaluating the differences between strata-specific estimates (age, sex, seasons, hospitalization or ERVs). Additional subgroup analyses were performed for short- (≤2 days) and long-lag (>2 days) patterns. The default for short-lag patterns was the most frequently used for individual pollutants; otherwise, lag1 or lag0 or lag 0–1 served as surrogates. For long-lag patterns, single lag was a priority selection compared with the cumulative lag.

Potential publication bias was assessed by using the asymmetric plot and confirmed by the Egger’s test [100].

Statistical analyses were conducted using STATA 11.2 (Stata, College Station, TX, USA). All tests were two-sided and statistical significance was defined as *P*<0.05, except for the heterogeneity assessment (*P*<0.10).

The PRISMA checklist for this meta-analysis could be found in **S1 File** in the supporting information.

## Results

### Eligible studies

**Fig 1:**
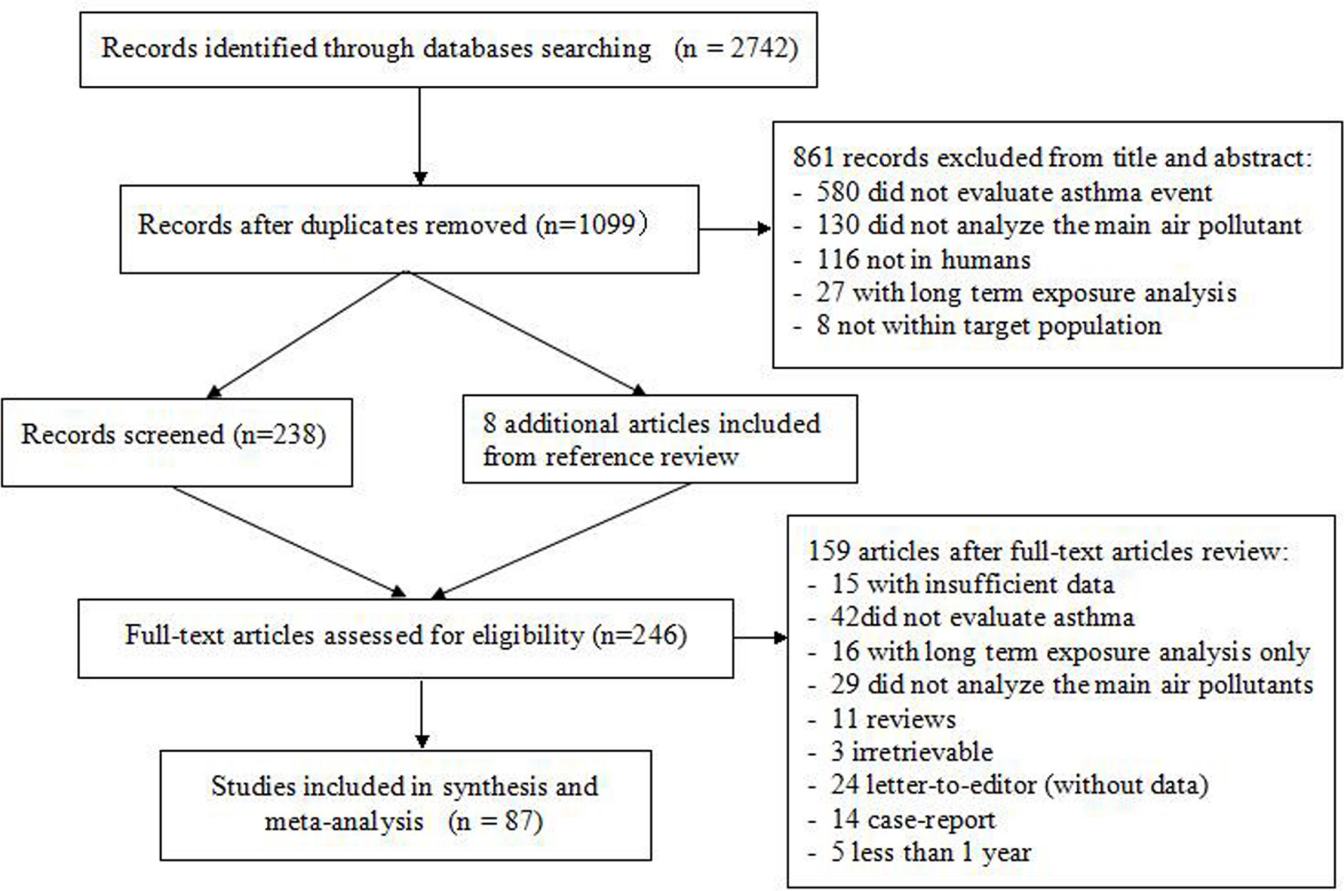
Study flow chart.

We initially identified 1099 literature reports. After screening for the titles and abstracts, 246 full-text articles were assessed for eligibility, of which 87 were included (86 in English and 1 in Spanish, **Fig 1**).

Main characteristics of 87 eligible studies, consisting of 62 time-series and 25 case cross-over studies, are displayed in S1 Table. Databases were extracted from 46 ERVs, 37 hospital admissions and 4 ERVs/hospital admissions. The study cohort consisted of the general population. Lag exposures varied from a specific day (single-lag) to 7 days or less before the onset of asthma exacerbations.

Of the 87 included studies, 50 focused on children, 21 on adults, 13 on elderly population, and 44 on general population. Only 12 studies have conducted sex modification analyses, with 12 male and 11 female sub-datasets. 31 studies were performed in warm seasons and 25 in cold seasons.

Mean 24-hr or 8-hr maximum concentrations of six pollutants are demonstrated in **S1 Table**.

### Overall analyses

**Fig 2:**
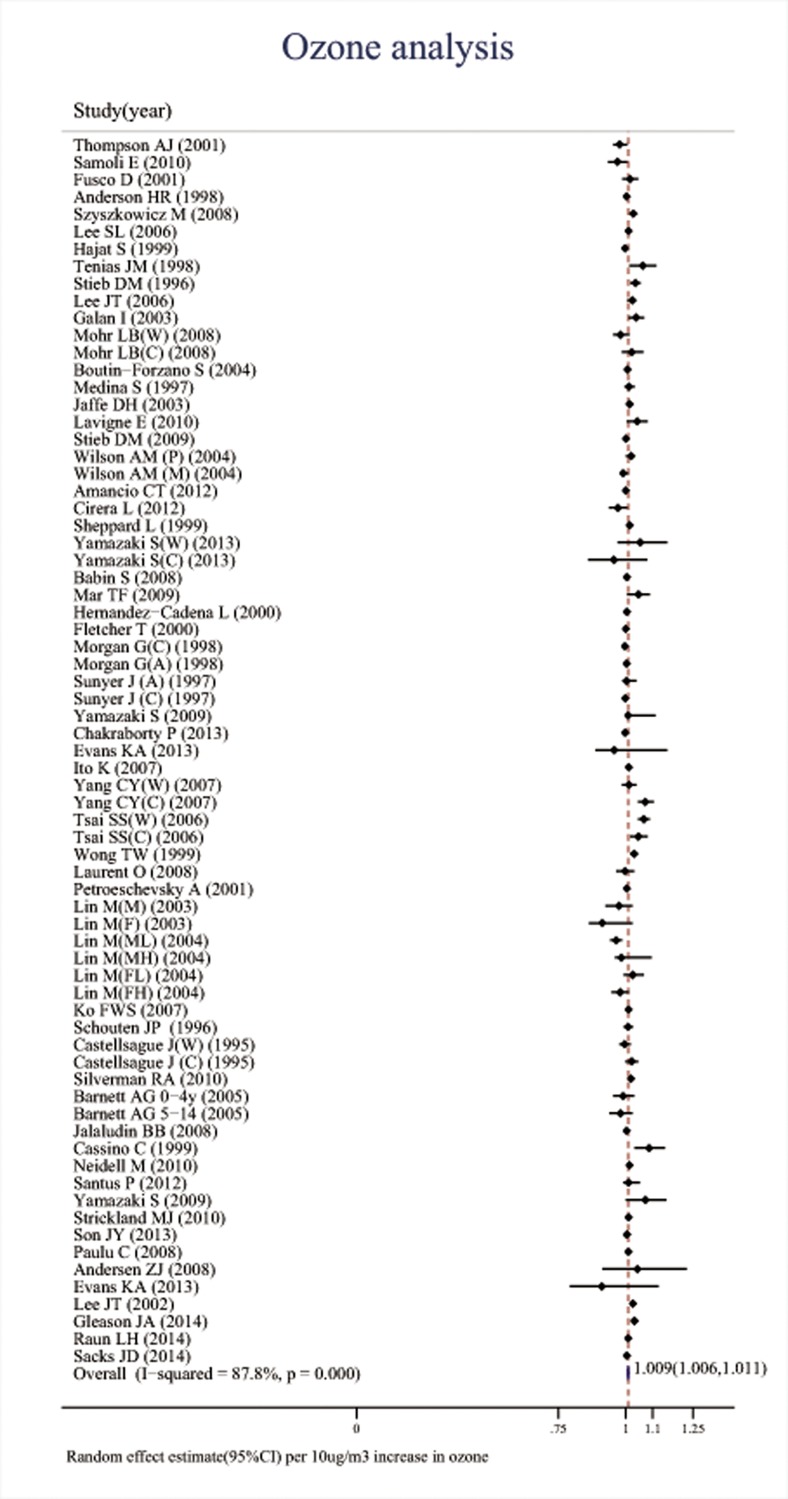
Association between ozone and ERVs/hospital admissions for asthma in the overall analyses.

**Fig 3:**
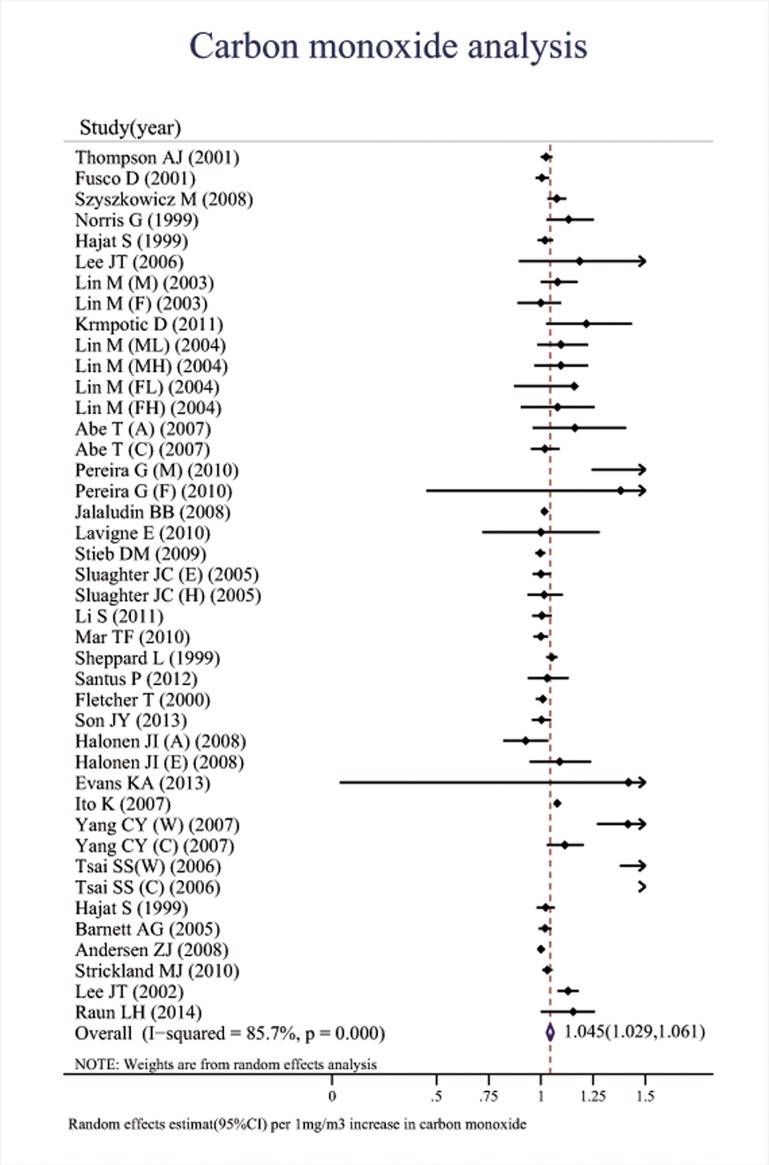
Association between carbon monoxide and ERVs/hospital admissions for asthma in the overall analyses.

**Fig 4:**
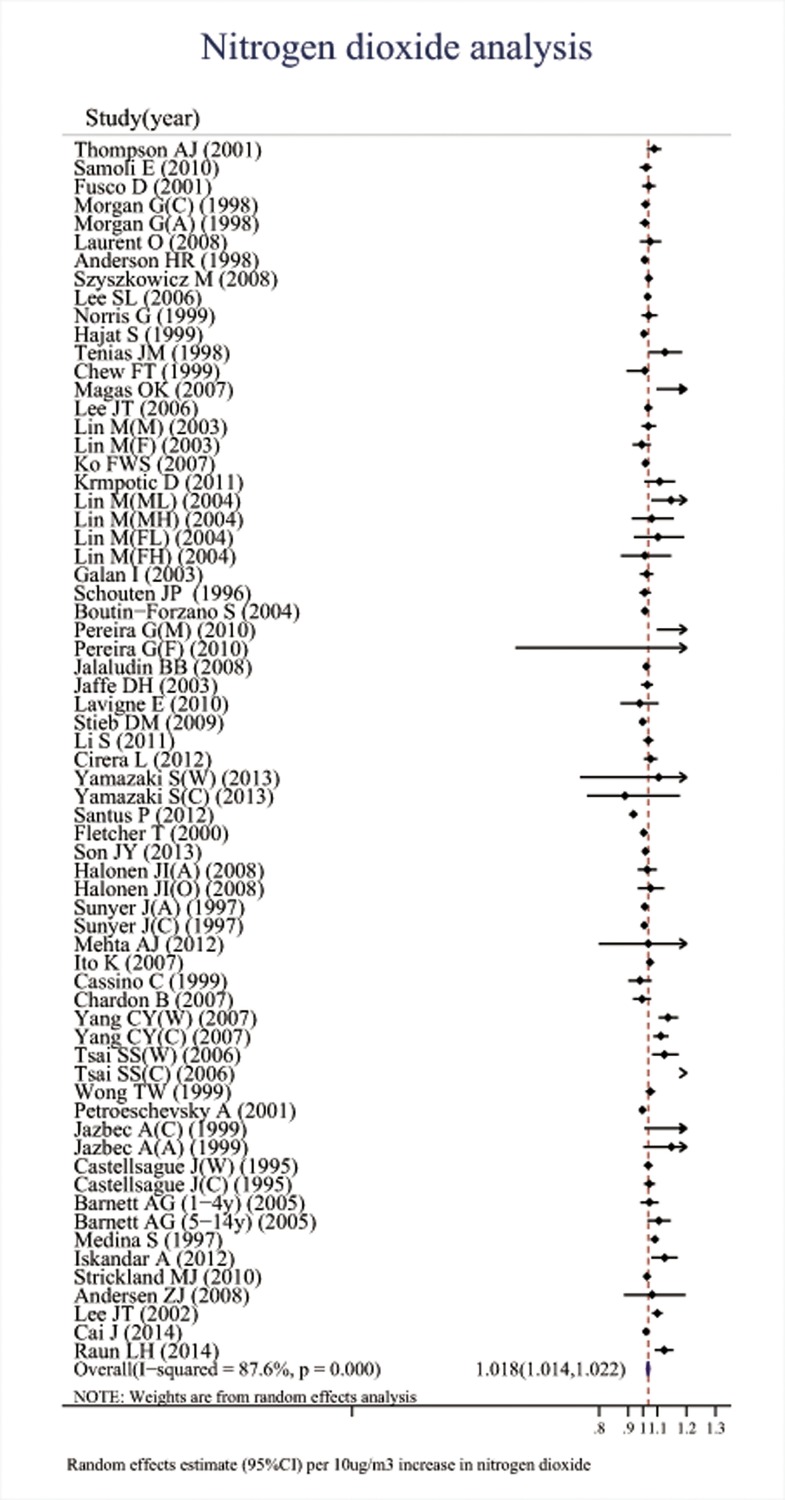
Association between nitrogen dioxide and ERVs/hospital admissions for asthma in the overall analyses.

**Fig 5:**
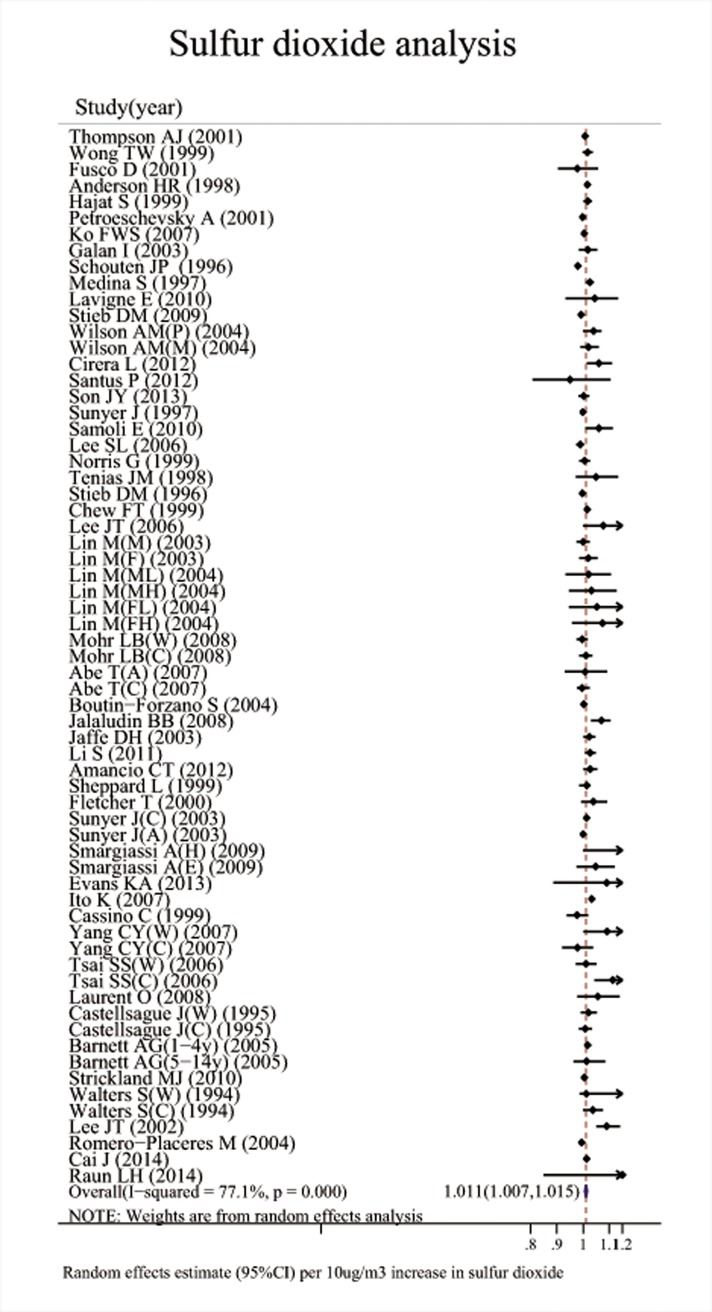
Association between sulfur dioxide and ERVs/hospital admissions for asthma in the overall analyses.

**Fig 6:**
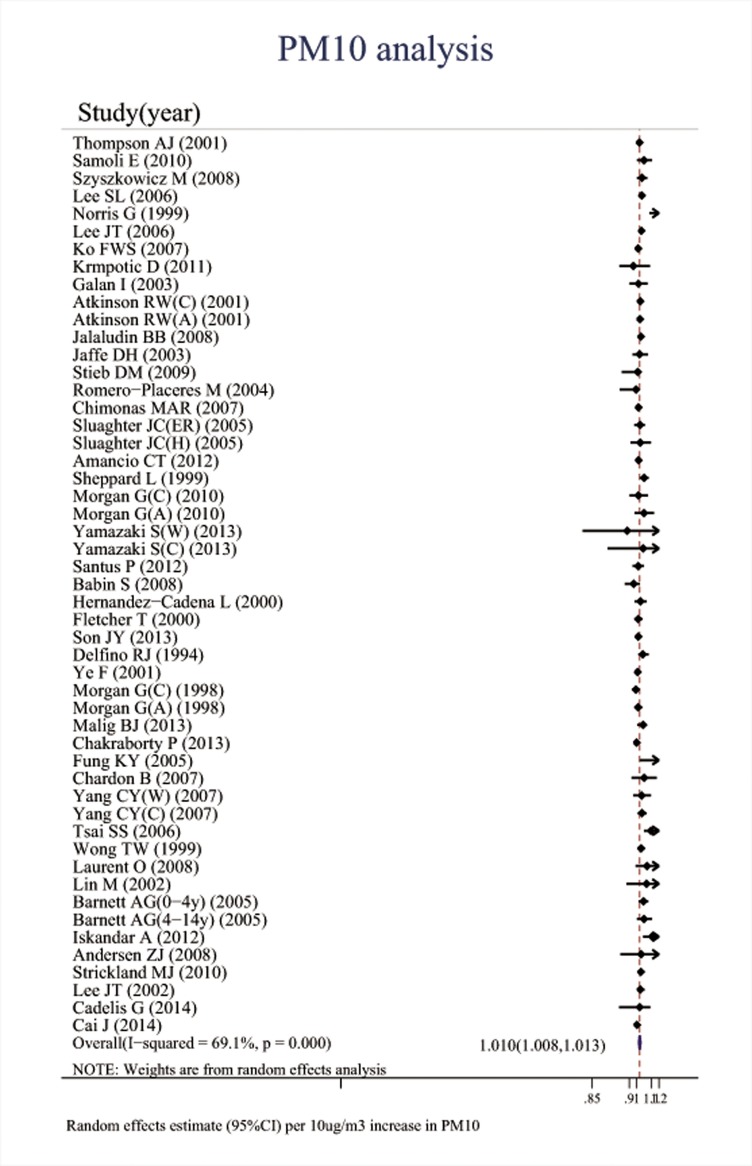
Association between PM_10_ and ERVs/hospital admissions for asthma in the overall analyses.

**Fig 7:**
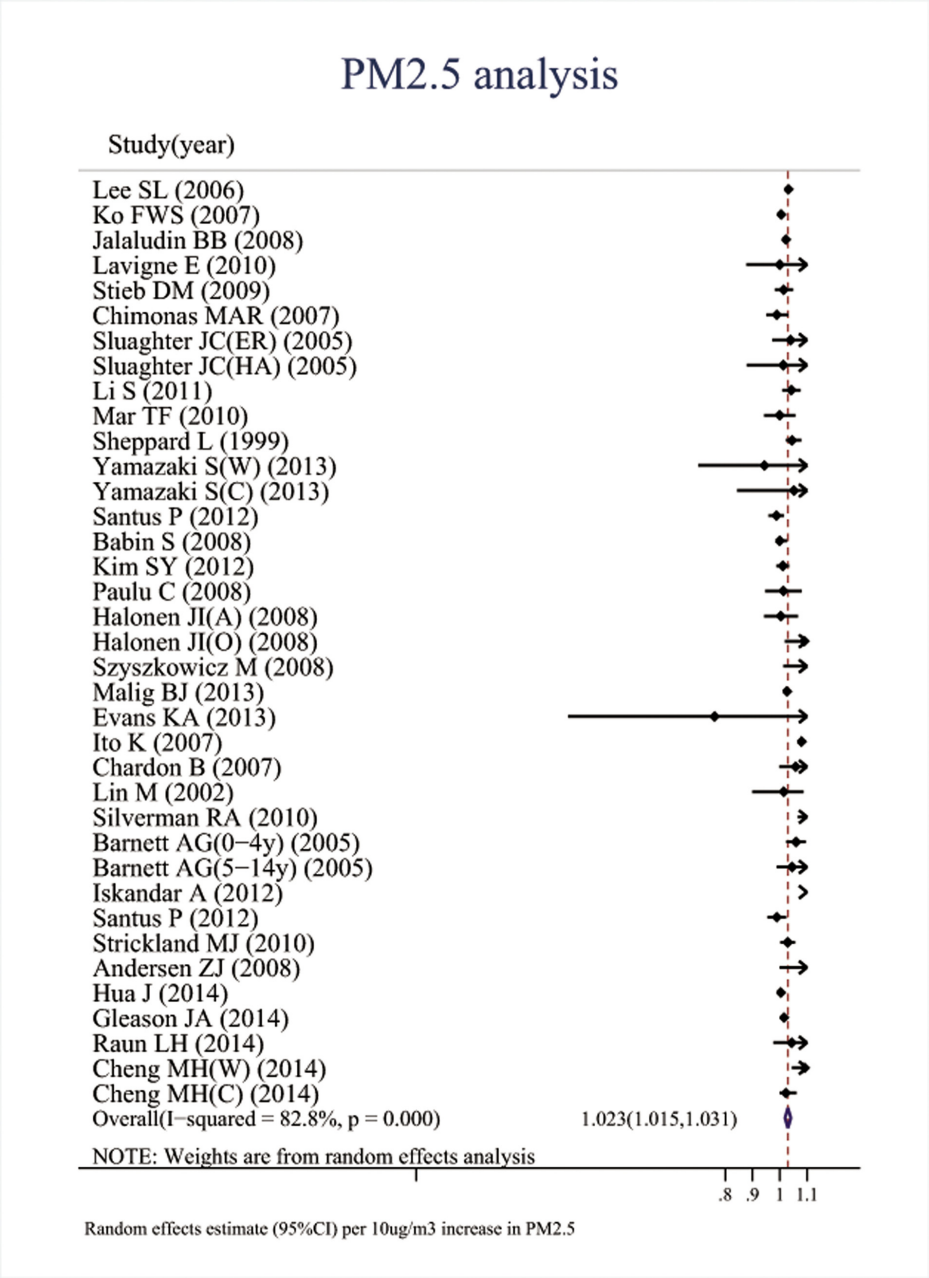
Association between PM_2.5_ and ERVs/hospital admissions for asthma in the overall analyses.

Associations between the six major air pollutants and asthma-related ERVs/hospitalizations were statistically significant [O_3_: 71 studies; RR (95%CI), 1.009 (1.006, 1.011); I^2^ = 87.8%, PAF (95%CI): 0.8 (0.6, 1.1); CO: 42 studies; RR (95%CI), 1.045 (1.029, 1.061); I^2^ = 85.7%, PAF (95%CI): 4.3 (2.8, 5.7); NO_2_: 66 studies; RR (95%CI), 1.018 (1.014, 1.022); I^2^ = 87.6%, PAF (95%CI): 1.8 (1.4, 2.2); SO_2_: 65 studies; RR (95%CI), 1.011 (1.007, 1.015); I^2^ = 77.1%, PAF (95%CI): 1.1 (0.7, 1.5); PM_10_: 51 studies; RR (95%CI), 1.010 (1.008, 1.013); I^2^ = 69.1%, PAF (95%CI): 1.1 (0.8, 1.3); PM_2.5_: 37 studies; RR (95%CI), 1.023 (1.015, 1.031); I^2^ = 82.8%, PAF (95%CI): 2.3 (1.5, 3.1)]. (**Table 1, Figs 2–7**).

**Table 1:** Association of six major air pollutants with ERVs and hospital admissions for asthma in overall and sensitivity analyses.

Characteristics	Air pollutant (incremental unit)					
	O_3_(10 μg/m^3^)	CO (1 mg/m^3^)	NO_2_ (10 μg/m^3^)	SO_2_ (10 μg/m^3^)	PM_10_ (10 μg/m^3^)	PM_2.5_ (10 μg/m^3^)
**Overall study analyses**						
**No. of the studies**	71	42	66	65	51	37
**I** ^**2**^ **,%**	87.8	85.7	87.6	77.1	69.1	82.8
**RR (95%CI)**	1.009 (1.006,1.011)	1.045(1.029, 1.061)	1.018 (1.014, 1.022)	1.011 (1.007, 1.015)	1.010 (1.008, 1.013)	1.023(1.015, 1.031)
**Egger’s test, P value**	<0.001	0.01	<0.001	<0.001	<0.001	0.06
**PAF, %(95%CI)** ^**a**^	0.8 (0.6, 1.1)	4.3 (2.8, 5.7)	1.8 (1.4, 2.2)	1.1 (0.7, 1.5)	1.1 (0.8, 1.3)	2.3 (1.5, 3.1)
**Study quality sensitivity analyses**						
**No. of the studies**	12	7	11	13	7	3
**I** ^**2**^ **,%**	55.7	13.6	55.7	57.9	0.0	0.0
**RR (95%)**	1.005 (1.002,1.008)	1.013 (1.000, 1.028)	1.009 (1.004, 1.015)	1.009 (1.003, 1.015)	1.006 (1.003, 1.009)	1.004 (1.000, 1.009)
**Egger’s test, P value**	0.15	0.16	0.16	0.28	0.53	0.71
**Lag exposures sensitivity analyses**						
**No. of the studies**	9	14	11	12	13	13
**Lag exposure, d**	0	1	0	0	1	1
**I** ^**2**^ **,%**	22.4	0.0	65.9	41.8	0.0	6.6
**RR (95%)**	1.010 (1.005, 1.014)	1.013 (1.001, 1.025)	1.010 (1.002, 1.018)	1.004 (1.000, 1.008)	1.005 (1.003,1.008)	1.008 (1.003, 1.013)
**Egger’s test, P value**	0.99	0.08	0.93	0.96	0.42	0.49

Abbreviations: PAF, population attributable fraction; PM_10_, particulate matter of ≤10 μm; PM_2.5_, particulate matter of ≤2.5 μm; RR, relative risk

The PAF was calculated by {PAF = P[(RR−1)/P(RR−1)+1]}, where P indicates prevalence of exposure to air pollution in the population which was estimated as 100%.

Publication bias was detected in all analyses evaluating all pollutants except for PM_2.5_ (P = 0.06). See S1 Fig in the online supplement for the funnel plots of the relative risks of emergency/hospital admissions for asthma in relation to six air pollutants in the overall analyses. The Excel form of our database (**S2 File**) is also available in the supporting information.

### Sensitivity analyses

#### Study quality

There were significant associations between air pollutants and asthma-related ERVs/hospitalizations in the sensitivity analyses, based on 29 studies fulfilling the quality score of 5 points without significant publication bias [O_3_: 12 studies; RR (95%CI), 1.005 (1.002, 1.008); I^2^ = 55.7%; Egger’s test, *P* = 0.15; CO: 7 studies; RR (95%CI), 1.013 (1.000, 1.028); I^2^ = 13.6%; Egger’s test, *P* = 0.16; NO_2:_ 11 studies; RR (95%CI), 1.009 (1.004, 1.015); I^2^ = 55.7%; Egger’s test, *P* = 0.16; SO_2:_ 13 studies; RR (95%CI), 1.009 (1.003, 1.015); I^2^ = 57.9%; Egger’s test, *P* = 0.28; PM_10_: 7 studies; RR (95%CI), 1.006 (1.003, 1.009); I^2^ = 0.0%; Egger’s test, *P* = 0.53; PM_2.5_: 3 studies; RR (95%CI), 1.004 (1.000, 1.009); I^2^ = 0.0%; Egger’s test, *P* = 0.71] (**Table 1, and S1 Fig** in the online supplement for the funnel plots of the relative risks of emergency/hospital admissions for asthma in relation to six air pollutants regarding studies with a score of 5.)

#### Lag exposure

**Fig 8:**
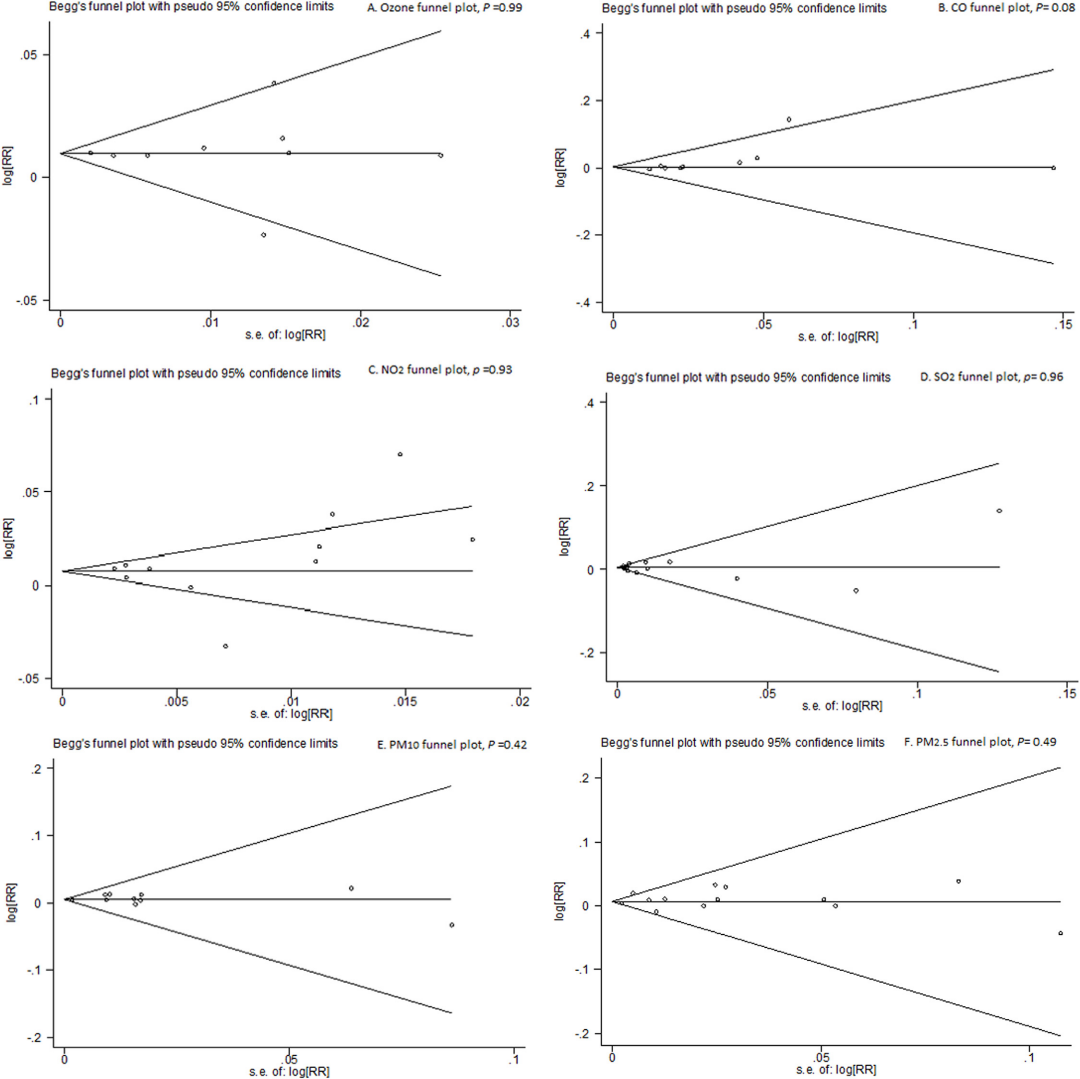
Funnel plots for relative risks of ERVs/hospital admissions for asthma in relation to the six air pollutants in lag pattern sensitivity analyses. A) Funnel plot for ozone; B) Funnel plot for CO; C) Funnel plot for NO_2_; D) Funnel plot for SO_2_; E) Funnel plot for PM_10_; F) Funnel plot for PM_2.5._

Lag exposure was 0 day for O_3_, NO_2_ and SO_2_, and 1 day for CO, PM_10_ and PM_2.5_. Likewise, associations between the six air pollutants and asthma-related ERVs/hospitalizations were statistically significant [O_3_: 9 studies; RR (95%CI), 1.010 (1.005, 1.014); I^2^ = 22.4%; Egger’s test, *P* = 0.99; CO: 14 studies; RR (95%CI), 1.033 (1.001, 1.025); I^2^ = 0.0%; Egger’s test, *P* = 0.37; NO_2:_ 11 studies; RR (95%CI), 1.010 (1.002, 1.018); I^2^ = 65.9%; Egger’s test, *P* = 0.93; SO_2:_ 12 studies; RR (95%CI), 1.004 (1.000, 1.008); I^2^ = 41.8%; Egger’s test, *P* = 0.96; PM_10_: 12 studies; RR (95%CI), 1.005 (1.003, 1.008); I^2^ = 0.0%; Egger’s test, *P* = 0.42; PM_2.5_: 13 studies; RR (95%CI), 1.008 (1.003, 1.013); I^2^ = 6.6%; Egger’s test, *P* = 0.49]. No publication bias was detected. (**Table 1, Fig 8**)

### Subgroup analyses

Effect modification of O_3_, CO, NO_2_, PM_10_ and PM_2.5_ on asthma-related hospital admission and ERVs (stronger association for hospital admissions) was found.

In subgroup analysis of sex, more pronounced associations were demonstrated in males [CO: 1.080 (1.047, 1.113), NO_2_:1.028 (1.008, 1.038); PM_10_: 1.025 (1.011, 1.039); PM_2.5_: 1.013 (1.000, 1.018)]. No significant association was found in females, except for exposure to O_3_ [1.023 (1.006, 1.040)].

There was a tendency towards stronger associations between ERVs/hospital admissions and the six air pollutants in children [CO: 1.018 (1.013, 1.023); NO_2_: 1.018 (1.013, 1.023); SO_2_: 1.016 (1.011, 1.022); PM_10_: 1.013 (1.008, 1.018); PM_2.5_: 1.025 (1.013, 1.037)] and the elderly [CO: 1.094 (1.002, 1.185); NO_2_: 1.019 (1.013, 1.024); SO_2_: 1.024 (1.005, 1.044)] as compared with the adults.

Stronger associations can also be observed in warm seasons [CO: 1.166 (1.099, 1.232), NO_2_: 1.029 (1.018, 1.040); SO_2_: 1.018 (1.010, 1.026); PM_10_: 1.021 (1.007, 1.023); PM_2.5_: 1.028 (1.011, 1.044)], except for ozone exposure.

Additional subgroup analyses demonstrated that long-lag patterns that were associated with significant heterogeneity yielded a stronger association than short-lag patterns (**Table 2**).

**Table 2:** Stratum-specific combined estimates of the association of six major air pollutants with ERVs and hospital admissions for asthma.

**Stratum**	Air pollutant (incremental unit)																	
	O_3_(10 μg/m^3^)			CO (1 mg/m^3^)			NO_2_ (10 μg/m^3^)			SO_2_ (10 μg/m^3^)			PM_10_ (10 μg/m^3^)			PM_2.5_ (10 μg/m^3^)		
	No.	RR (95%CI)	I^2^	No.	RR (95%CI)	I^2^	No.	RR (95%CI)	I^2^	No.	RR (95%CI)	I^2^	No.	RR (95%CI)	I^2^	No.	RR (95%CI)	I^2^
**Sex**																		
**Males**	9	1.009 (0.993,1.025)	77	7	1.080 (1.047,1.113)	0	10	1.028 (1.008,1.038)	68	7	1.018 (0.996,1.040)	40	8	1.025 (1.011,1.039)	80	5	1.013 (1.000,1.018)	79
**Females**	9	1.023 (1.006,1.040)	79	7	1.045 (0.966,1.124)	33	10	1.008 (0.987,1.029)	71	7	1.014 (0.996,1.021)	0	6	1.005 (1.000,1.009)	0	4	1.012 (1.000,1.020)	7
**Age**																		
**Children**	42	1.008 (1.005,1.012)	89	29	1.018 (1.013,1.023)	70	39	1.018 (1.013,1.023)	87	37	1.016 (1.011,1.022)	53	25	1.013 (1.008,1.018)	83	20	1.025 (1.013,1.037)	82
**Adults**	23	1.013 (1.008,1.018)	79	7	1.004 (0.963,1.045)	13	14	1.008 (1.003,1.014)	68	15	1.002 (0.997,1.007)	26	7	1.009 (1.003,1.014)	29	6	1.027 (1.007,1.047)	56
**Elderly**	10	1.010 (1.002,1.017)	61	5	1.094 (1.002,1.185)	40	9	1.019 (1.013,1.024)	0	9	1.024 (1.005,1.044)	25	6	1.009 (1.004,1.015)	52	5	1.022 (1.014,1.031)	0
**Season**																		
**Cold**	22	1.017 (1.007,1.028)	82	10	1.087 (1.020,1.154)	94	21	1.026 (1.016,1.038)	85	19	1.012 (1.002,1.022)	71	11	1.014 (1.006,1.023)	71	13	1.004 (0.997,1.011)	61
**Warm**	29	1.016 (1.010,1.021)	87	12	1.166 (1.099,1.232)	85	23	1.029 (1.018,1.040)	84	22	1.018 (1.010,1.026)	43	14	1.021 (1.007,1.023)	74	15	1.028 (1.011,1.044)	91
**Type of admission**																		
**ERV**	40	1.007 (1.004,1.010)	85	22	1.030 (1.011,1.049)	81	33	1.013 (1.008,1.018)	73	34	1.013 (1.007,1.019)	74	23	1.010 (1.006,1.014)	65	23	1.017 (1.006,1.029)	77
**HA**	31	1.010 (1.006,1.014)	84	20	1.080 (1.048,1.112)	87	33	1.023 (1.017,1.029)	91	31	1.008 (1.002,1.014)	71	28	1.011 (1.008,1.015)	70	14	1.028 (1.017,1.040)	85
**Lag pattern**																		
**Lag ≤2days**	49	1.007 (1.004,1.011)	82	32	1.036 (1.018,1.055)	78	44	1.013 (1.009,1.014)	70	48	1.008 (1.004,1.013)	73	35	1.008 (1.005,1.010)	67	27	1.021 (1.011,1.031)	83
**Lag >2days**	23	1.010 (1.006,1.013)	90	11	1.082 (1.042,1.122)	92	22	1.028 (1.019,1.036)	92	17	1.022 (1.012,1.032)	79	16	1.016 (1.012,1.020)	4	10	1.031 (1.012,1.050)	78

## Discussion

### Principal findings

We have systematically evaluated and confirmed the associations between short-term exposure to six air pollutants which are closely regulated by the environmental protection agencies and asthma-related ERV/hospitalizations, based on all available time-series and case-crossover studies. Low heterogeneity and no publication bias was observed in the sensitivity analyses. Our findings remained robust, despite potential publication bias resulting from the relatively small sample sizes in sensitivity analyses.

### Mechanisms of air pollutants on eliciting asthma exacerbations

The observed effects of the six major air pollutants on asthma ERVs/hospitalization are biologically plausible. The major mechanisms of individual air pollutants responsible for triggering asthma exacerbations are as follows:

** NO_2_ and ozone have been implicated in eliciting lipid peroxidation of the cell membranes and the generation of various free radicals which collectively impair the structure and function of the asthmatic airways [101]. Furthermore, exposure to ozone and (or) NO_2_ can also promote the release of inflammatory mediators (i.e. interleukin-8, granulocyte macrophage-colony stimulating factor [102],

** SO_2_, a well-known inorganic chemical irritant, has been demonstrated to promote airway inflammation (increased levels of tumor growth factor-β in bronchoalveolar fluids) and eosinophilia, induce bronchospasm and airway fibrosis (a factor potentially leading to increased airway responsiveness) in asthma [103].

** Particulate matters harbor a more complex impact on asthmatic airways, since their deposition in the airways directly elicited airway inflammation, mucosal edema and cytotoxicity [104]. The convergence of regulatory signals generated by particulate matter-induced oxidative stress in dendritic cells and their interactions may also be responsible for asthma exacerbations [105]. Furthermore, the defective airway macrophage phagocytosis, resulting from increased prostaglandin E_2_ levels, could have augmented the adverse effects of inhaled carbonaceous particulate matters on eliciting exacerbations [106].

** The direct association between CO and asthma is unfortunately less clearer [29,107]. It is plausible that CO might act as a surrogate of other noxious gases which are derived from incompletely combusted products. Moreover, CO seemed to confer greater adverse impacts on asthmatic children because of their immature lung development.

### Interpretation

Most studies were limited at all age intervals, which constituted an important effect modifier [52]. In keeping with literature reports [16,24,30,38,57], we further confirmed that children and elderly people were more susceptible to asthma exacerbations. A plausible interpretation could be that children harbor immature lung growth and host-defense capacity and that, in elderly individuals, air pollution amplifies inflammatory responses of remodeled airways.

To date, effect modification by sex has not been well established [5,7,37–38,40,50,68]. The higher rate of asthma-related hospitalizations and ERVs in males could not justify the sex-related susceptibility, since the exposures (outdoor occupations, social activities) and biologic characteristics (i.e. hormonal levels, lung size and growth and airway inflammation) are different. Furthermore, greater effects of ozone on females were consistent with those in a previous report [108].

If the additive effects of air pollution were season-independent, then the PAFs and RRs would be higher during warm seasons because of fewer competing pollutants. However, unlike other pollutants, the association of ozone (a component of “photochemical cocktail” which is typically a warm-season pollutant [24,27,31–32,52,54,76,78,82,87]) and asthma exacerbations was similar between the warm and cold seasons. Unfortunately, the variability of temperature adjustment approaches and the lack of information regarding solar radiation and brightness have constrained our analyses in determining the seasonal modifications. Furthermore, limiting the analysis to the above-mentioned confounders might have minimized the number of eligible studies, possibly resulting in inaccurate conclusions.

### Clinical significance

Despite the weak associations between air pollution and asthma exacerbations, the effects of air pollution were globally considerable because the RRs and percentage increase were derived from large cohorts in time-series studies, reflecting significant healthcare utilization, immense social and economical burden. Despite that the high PAFs for outdoor air pollution was essentially imputed from the prevalence of exposure of 100%, this assumption may still be reasonable, since epidemiological studies generally assigned outdoor average level to all individuals. Furthermore, we quantified asthma risk according to the changes in air pollution, since asthma-related ERVs/hospitalizations and the changes in air pollutant concentrations would assume a linear correlation, and hence, no positive threshold [109] could be established.

### Limitations

First, the differences between ERVs and hospitalizations did have certain impacts on their utility for quantifying the observed associations with air pollution in subgroup analysis. However, a high degree of heterogeneity could be observed in analyses of all strata, including hospital admissions and ERVs. This might be linked to the various study design quality, inclusion criteria, analytic strategies and lag patterns. Heterogeneities were reduced dramatically among studies with a common analytic strategy (most commonly used lag patterns) or standardized protocol (study quality >5), highlighting the importance of standardized study protocol with the most appropriate lag.

Second, we did not analyze the association between air pollutants and other systemic diseases, therefore the multi-faceted adverse effects of air pollution could have been markedly diluted. Lower levels of air pollutants reportedly led to attenuated asthma symptoms [110–111], airway inflammation [112], lung function improvement [112] and less healthcare utilization and access to medications [113], confirming the roles of air pollution on eliciting asthma exacerbations.

Third, the coefficients from “single-pollutant” model were utilized despite potential interactions among different air pollutants. Regarding that the lack of crystal-clear exposure-asthma relationships hampered selection of additive or multiplicative model, and that a large number of complex parameters rendered the ideal ‘multivariate’ meta-analysis computationally impractical [114], we therefore independently analyzed the effects of individual pollutants [15, 17–18, 115].

Finally, the methodologies of lag selection remain controversial. Any particular lag selection would have excluded a considerable number of studies. In this study, we chose the most frequently used short lags (lag0, lag1 or lag0-1), since longer lags have been less consistently reported in previous literature and harbored a significant heterogeneity in our pooled analysis.

### Practical implications

Our findings has called for the implementation of more stringent regulations on the traffic and industry, including the utilization of environmental-friendly fuels (i.e. liquid natural gas, diesel derived from biomass fuels, hydrogen gas), engines or techniques (such as hybrid vehicles, purely electric motors), and the utilization of filters or absorbers of noxious gases before release into the atmosphere, and the upgrading of traditional industrial facilities (i.e. cement production). The development of fine particle separating facial masks or intranasal gel might be useful for the patients who have difficulty in avoiding direct exposure to exposure, particularly at the workplace. The levels of air pollutants should also be incorporated into weather forecasts so as to issue alerts to population at risk, thus facilitating administration of preventative medications.

## Conclusion

Short-term exposure to air pollutants confers an increased risk of asthma-related ERVs and hospital admissions. Our findings call for greater awareness of environmental protection and the implementation of effective measures to improve the quality of air, which may reduce the risks of adverse effects on the population’s health. However, the effects need to be interpreted cautiously since longer lags are essential in time-series studies to better determine the effects of outdoor air pollution on asthma outcomes.

## Supporting Information

S1 Fig Funnel plots of the relative risks of ERVs/hospital admissions for asthma in relation to six air pollutants in the overall analyses and sensitivity analyses (studies with a score of 5). Fig A. Funnel plot of the relative risks of ERVs/hospital admissions for asthma in relation to CO in the overall analyses; Fig B. Funnel plot of the relative risks of ERVs/hospital admissions for asthma in relation to NO_2_ in the overall analyses; Fig C. Funnel plot of the relative risks of ERVs/hospital admissions for asthma in relation to ozone in the overall analyses; Fig D. Funnel plot of the relative risks of ERVs/hospital admissions for asthma in relation to PM_2.5_ in the overall analyses; Fig E. Funnel plot of the relative risks of ERVs/hospital admissions for asthma in relation to PM_10_ in the overall analyses; Fig F. Funnel plot of the relative risks of ERVs/hospital admissions for asthma in relation to SO_2_ in the overall analyses; Fig G. Funnel plot of the relative risks of ERVs/hospital admissions for asthma in relation to CO in the sensitivity analyses regarding studies with a score of 5; Fig H. Funnel plot of the relative risks of ERVs/hospital admissions for asthma in relation to NO_2_ in the sensitivity analyses regarding studies with a score of 5; Fig I. Funnel plot of the relative risks of ERVs/hospital admissions for asthma in relation to ozone in the sensitivity analyses regarding studies with a score of 5; Fig J. Funnel plot of the relative risks of ERVs/hospital admissions for asthma in relation to PM_2.5_ in the sensitivity analyses regarding studies with a score of 5; Fig K. Funnel plot of the relative risks of ERVs/hospital admissions for asthma in relation to PM_10_ in the sensitivity analyses regarding studies with a score of 5; Fig L. Funnel plot of the relative risks of ERVs/hospital admissions for asthma in relation to SO_2_ in the sensitivity analyses regarding studies with a score of 5.
(EPS)

S1 File PRISMA checklist. (DOC)

S2 File The database of the association between six air pollutants and ERVs/hospital admissions for asthma in overall analyses (in Excel form). (XLS)

S1 Table Search strategies and main characteristics of all included studies. (DOC)
