# Supplementary material for: Association between Air Pollutants and Asthma Emergency Room Visits and Hospital Admissions in Time Series Studies: A Systematic Review and Meta-Analysis
Source: PLoS One. 2015 Sep 18;10(9):e0138146. doi: 10.1371/journal.pone.0138146 (PMC4575194; doi:10.1371/journal.pone.0138146)
Supplement: S1 Table — (DOC) [file pone.0138146.s004.doc]

**E-Appendix: Search strategies**

Database(s): EMBASE 1988 to May 2014, Ovid MEDLINE In-process & Other Non-indexed Citations and Ovid MEDLINE 1948 to present, EBM Reviews-Cochrane Central Register of Controlled Trials, EBM Reviews-Cochrane Database of Systematic Reviews 2005 to May 2014.

| **﹟** | **Search strategies** | **Results** |
| --- | --- | --- |
| 1 | exp Asthma/ | 182357 |
| 2 | exp Wheeze | 13635 |
| 3 | exp ‘respiratory tract allergy’ | 213770 |
| 4 | Or 1-3 | 219475 |
| 5 | exp air pollution/ | 114335 |
| 6 | exp Particulate Matter/ | 23190 |
| 7 | ((air or atmosphere or atmospheric) adj (pollution * or polluted or pollutant* or contamination or contaminated)).mp. [mp=ti, ab, sh, hw, tn, ot, dm, mf, ps, rs, nm, ui, kw, tx, ct] | 156170 |
| 8 | (‘particulate matter’ or PM2.5 or PM10).mp. [mp=ti, ab, sh, hw, tn, ot, dm, mf, ps, rs, nm, ui, kw, tx, ct] | 32864 |
| 9 | exp ozone/ | 20388 |
| 10 | exp Carbon Monoxide/ | 27567 |
| 11 | exp Carbon Monoxide intoxication / | 5042 |
| 12 | exp nitrogen dioxide/ | 7833 |
| 13 | exp Sulfur Dioxide/ | 12095 |
| 14 | (ozone or ‘O_3_’ or ‘carbon monoxide’ or carbon monoxide or ‘CO’ or ‘nitrogen dioxide’ or ‘NO_2_’ or ‘N_2_O_4_’ or ‘dinitrogen tetraoxide’ or ‘nitrogen peroxide’ or ‘nitrogen tetraoxide’ or nitrogen oxide or ‘nitrous dioxide’ or ‘sulphur dioxide’ or ‘sulphur dioxide’ or ‘sulfurous anhydride’ or ‘SO_2_’).mp. [mp=ti, ab, sh, hw, tn, ot, dm, mf, ps, rs, nm, ui, kw, tx, ct] | 1078084 |
| 15 | Or 5-14 | 1214094 |
| 16 | #4 and #15 | 6274 |
| 17 | limit 16 to human [Limit not valid in CCTR,CDSR; records were retained] | 2742 |
| 18 | remove duplicates from 17 | 1091 |

**Table S1. Main characteristics of the included studies**

| **Study** | **Location/Period** | **Quality score** | **Sample size** | **Population** | **Diagnosis standard** | **Type of study** | | **Pollutants and concentration** | | | | | | **Measurement quality**  **score (0-1 point)** | | | **Lag pattern(single day/ mean days)** | | **Adjustments (long-term trend, seasonality, temperature, humidity, pressure, day for the week, holiday and influenza epidemics )** |
| --- | --- | --- | --- | --- | --- | --- | --- | --- | --- | --- | --- | --- | --- | --- | --- | --- | --- | --- | --- |
|  |  |  |  |  |  |  | | **CO** | **PM_10_** | **PM_2.5_** | **SO_2_** | **NO_2_** | **Ozone** |  | |  | |  | |
| **Studies with emergency room visit indices** | | | | | | | | | | | | | | | | | | | |
| Thompson AJ^3^  2001 | Belfast, Northern Ireland/  3 yr | 5 | - | Children | - | TS | | **0.65** | 28.4 | - | 47.1 | 43.6 | 38.4 | 1 | | Both | | long-term trend, seasonality, temperature, humidity, day of week, public holiday | |
| Sunyer J^14^  1997 | Barcelona, Helsinki, Paris, London, 6yr | 5 | 75190 | Children  + adults | ICD-9: 493 | TS | |  |  |  | 27.8 | 49.8 | 43.8 | 1 | | Both | | long-term trend, seasonality, temperature, humidity, day-of-week, influenza epidemics | |
| Chardon B^15^  2007 | Paris, 3yr | 5/4^b^ | 8027 | General | ICPC2.R96 | TS | |  | 23.0 | 14.7 |  | 44.4 |  | 1/0^b^ | | Cumulative | | long-term trends, seasonality, temperature, humidity, day-of-week, influenza epidemic, pollen | |
| Halonen JI^16^  2008 | Helsinki, Finland, 6 yr | 5 | 4807 | General | ICD-9: 493 | TS | | 0.5^a^ |  | 9.5 |  | 28.2 |  | 1 | | Single | | long-term trends, seasonality, temperature, humidity, day-of-week, public holiday, influenza epidemics | |
| Hajat S^30^  1999 | London,UK, 3yr | 5 | 38653 | General | ICD-9 J493 | TS | | 1.0 | 28.5 |  | 21.2 | 69.0 | 37.5 | 1 | | Both | | Long term trend, seasonality,  temperature, humidity, day-of-week, influenza epidemic | |
| Galan I^41^  2003 | Madrid, Spain, 3yr | 5 | 4827 | General | ICD-9 J493 | TS | |  | 32.1 |  | 23.6 | 67.1 | 45.8 | 1 | | Single | | Long-term trend, seasonality, temperature, humidity, pressure, day-of-week, public holiday, influenza epidemics | |
| Atkinson RW^51^  2001 | Barcelona, Birmingham, London, Milan, the  Netherlands, Paris, Rome, and Stockholm, 4yr | 5 | - | General | ICD-9 J493 | TS | |  | 29.3 |  |  |  |  | 1 | | Single | | long-term trend, seasonality, temperature, humidity, day for the week, holiday and influenza epidemics | |
| Medina S^55^  1997 | Paris, France, 4yr | 5 | - | General | SOS-Medecins R01 | TS | |  |  |  | 19.0 | 56.0 | 34.0 | 1 | | Both | | long-term trend, seasonality, temperature, humidity, day-of-week, influenza epidemics | |
| Stieb DM^58^  2009 | Canada(Montreal, Ottawa, Edmonton, Saint John, Halifax, Toronto, Vancouver), 10yr | 5/4 ^b^ | 83563 | General | ICD-9/10 493;  J45 | TS | | 0.8 | 8.3 | 20.6 | 14.6 | 37.6 | 40.0 | 0/1^a^ | | Single | | long-term trend, seasonality, temperature, humidity, day-of-week, holiday | |
| Wilson AM^60^  2005 | Portland, Maine, Manchester, New Hampshire, 2yr | 5 | 7300 | General | ICD-9 J493 | TS | |  |  |  | 39.4^b^ |  | 41.0 | 1/0(ozone) | | Single | | long-term trend, seasonality, temperature, humidity, day-of-week, influenza epidemic | |
| [Sunyer J](http://www.ncbi.nlm.nih.gov/pubmed?term=Sunyer Deu J[Author]&cauthor=true&cauthor_uid=9864900)^77^  2003 | Birmingham, London, Milan, the Netherlands, Par is, Rome, Stockholm, 2-8 yr | 5 | - | Children  + adults | ICD-9 J493 | TS | |  |  |  | 17.6 |  |  | 1 | | Single | | long-term trend, seasonality, temperature, humidity, day-of-week, holiday, influenza epidemic | |
| Szyszkowicz M^27^ 2008 | Edmonton, Canada, 10yr | 4 | 62563 | General | ICD-9 J493 | TS | | 0.9 | 22.6 | 8.5 |  | 45.0 | 39.9 | 1 | | Single | | Long term trend, temperature, seasonality  humidity, day-of-week | |
| Norris G^29^  1999 | Seattle, US, 27mon | 4 | 1458 | Children | ICD-9 J493 | TS | |  | 21.7 |  | 17.1 | 41.5 |  | 1 | | Single | | Long term trend, seasonality  temperature, humidity, day-of-week | |
| Tenias JM ^31^  1998 | Valencia, Spain, 3 yr | 4 | 734 | Adults + elderly | - | TS | |  |  |  | 26.6 | 57.7 | 62.8 | 1 | | Single | | long-term trend, seasonality, temperature, humidity, day-of-week, public holiday, influenza epidemics | |
| Stieb DM ^34^  1996 | Saint John,  New Brunswick, Canada, 8yr | 3 | 1163 | General | - | TS | |  |  |  |  |  | 89.1 | 1 | | Single | | long-term trend, seasonality, temperature, humidity, day-of-week, | |
| Mohr LB ^43^  2008 | St. Louis, US, 2yr | 3 | 12836 | Children | ICD-9 J493 | TS | |  |  | - |  | - | - | 1 | | Single | | Long-term trend, seasonality, temperature, | |
| Castellsague J ^46^  1995 | Barcelona, Spain, 5yr | 3 | 6019 | Adults+ elderly | - | TS | |  |  |  | 45.5 | 58.0 | 70.5 | 1 | | Cumulative | | Long term trend, seasonality, temperature, humidity, day-of-week, influenza epidemic | |
| Chakraborty P^47^  2013 | Kolkata city, India,  2yr | 4 | 2703 | Children | ICD-9 J493+ICD-10 J45/4d | TS | |  | 3.8 |  |  |  | 1.3^c^ | 1 | | - | | Long term trend, seasonality, temperature, humidity | |
| Jaffe DH^56^  2003 | Cincinnati, Cleveland, and  Columbus, US, 5yr | 4 | 4416 | Children+ adults | ICD-9 J493 | TS | |  | 49.5 |  | 28.8 | 10.1 | 35.8 | 1 | | Single | | long-term trend, seasonality, temperature, humidity, day-of-week | |
| Mar TF ^64^  2010 | Tacoma, Washington, 3.5 yr | 3 | 10091 | General | - | TS | | 1.2 |  | 12.3 |  |  |  | 1 | | Single | | long-term trend, seasonality, temperature, humidity | |
| Cirera L ^65^  2012 | Cartagena, Spain, 4yr | 4 | 1617 | General | - | TS | |  |  |  | 32 | 51 | 81 | 1 | | Single | | long-term trend, seasonality, temperature, humidity, day-of-week, public holiday, influenza epidemic | |
| Cassino C ^66^  1999 | New York, US, 3.5yr | 4 | 285 | Adults | NAEPP/ ICD-9 J493-493.9 | TS | | 1.2 |  |  | 29.4 | 92.4 | 37.5^c^ | 1 | | Single | | long-term trend, seasonality, temperature, humidity, day-of-week | |
| Babin S ^77^  2008 | Washington DC, US, 11yr | 4 | 61218 | General | ICD-9 J493 | TS | |  | - | - |  |  | - | 1 | | Single | | long-term trend, seasonality, temperature, humidity, day-of-week | |
| Mar TF ^79^  2009 | Seattle, Washington, US, 4yr | 3 | 3217 | Children  +  adults | ICD-9 J493-493.9 | TS | |  |  |  |  |  | 84.0 | 0 | | Single | | long-term trend, seasonality, temperature, humidity, day-of-week | |
| Hernandez-Cadena L ^81^  2000 | Ciudad Juárez, Chihuahua, Mexico, 1yr | 4 | 2459 | General | ICD-9 J493 | TS | |  | 34.46 |  |  |  | 110.6 | 1 | | Both | | long-term trend, seasonality, temperature, humidity, day-of-week | |
| Ito K^87^  2007 | New York, US,  4yr | 3 |  | General | - | TS | | 1.6^a^ | 15.7 |  | 22.3 | 31.1 | 65.1 | 1 | | Both | | long-term trend, seasonality, temperature, humidity, day-of-week | |
| Cadelis G^82^  2014 | Guadeloupe, France,  1yr | 3 | 836 | Children | ICD10: J45-J46 | TS | |  | 19.2 |  |  |  |  | 1 | | Both | | long-term trend, seasonality, temperature | |
| Jazbec A ^42^  1999 | Zagreb, Croatia, 1.5yr | 0 | 1372 | Children +adults | - | TS | |  |  |  |  | 45.1 |  | 0 | | Cumulative | | long-term trend, seasonality, | |
| Abe T ^48^  2007 | Tokyo, Japan, 1 yr | 2 | 6447 | General | - | TS | | 1.4 |  |  | 15.1 |  |  | 1 | | Single | | seasonality, temperature, humidity | |
| Chimonas MAR ^61^  2007 | Anchorage, Alaska, 3.5yr | 2 | 11037 | Children | ICD-9 J493 | TS | |  | 27.6 | 6.1 |  |  |  | 0 | | Single | | long-term trend, seasonality, temperature | |
| Strickland MJ^4^  2010 | Atlanta, US, 11 yr | 5 | 91386 | Children | ICD-9 J493 | CC | | 1.1^b^ | 23.8 | 16.4 | 30.9^b^ | 47.8^b^ | 97.3 | 1 | | Both | | long-term trend, seasonality, temperature, humidity, day of week and influenza epidemics | |
| Paulu C ^11^  2008 | Maine, US, 4yr | 5 | 8020 | General | ICD-9: 493 | CC | |  |  | 8.5 |  |  | 83.6 | 1 | | Both | | long-term trend, seasonality, temperature, humidity, day of week, holiday | |
| Jalaludin BB^54^  2008 | Sydney ,Australia, 5 yr | 5 | 317724 | Children | ICD-9 J493 | CC | | 1.0 | 16.8 | 9.4 | 3.1 | 47.6 | 67.7 | 1 | | Both | | long-term trend, seasonality, temperature, humidity, day-of-week, public holiday | |
| Lavigne E^57^  2010 | Windsor, Canada, 7 yr | 5 | 3728 | General | ICD-10: J45, J46, T486, Y556, Z825 | CC | | 0.4 |  | 7.3 | 5.3 | 17.4 | 41.5 | 1 | | Single | | long-term trend, seasonality, temperature, humidity, day-of-week, influenza epidemic | |
| Malig BJ ^22^  2013 | California,  4yr | 4 | 74978 | General | ICD-9 J493 | CC | |  | 35.0 | 12.1 |  |  |  | 1 | | Single | | long-term trends, seasonality, temperature, humidity, day of week | |
| Boutin-Forzano S^48^  2004 | Marseille, France, 1yr | 3 | 549 | Children + adults | - | CC | |  |  |  | 22.5 | 34.9 | 50.1 | 1 | | Single | | long-term trend, seasonality, temperature, humidity | |
| Yamazaki S ^72^  2013 | Himeji, Japan, 2yr | 3 | 956 | Children | - | CC | |  | 34.3 | 21.2 |  | 22.9 | 55.3 | 1 | | Single | | long-term trend, seasonality, temperature, humidity, day-of-week | |
| Santus P ^75^  2012 | Milan,  Italy, 2yr | 3 | 3569 | General | - | CC | | 1.5 | 47.1 | 32.8 | 4.13 | 102.6 | 74.3 | 1 | | Both | | long-term trend, seasonality, temperature, humidity, day-of-week | |
| Yamazaki S ^77^  2009 | Tokyo, Japan, 1yr | 4 | 403 | Children+ adults | - | CC | |  |  | 19.1 |  | 45.3 | 60.2 | 1 | | Cumulative | | long-term trend, seasonality, temperature, humidity, day-of-week, holiday | |
| Mehta AJ ^6^  2012 | Switzerland,  2yr | 1 | 147 | adults | - | CC | |  |  |  |  | 33.9 |  | 1 | | Single | | Long-term trend, influenza epidemics | |
| Laurent O ^23^  2008 | SMA, France 5yr | 2 | 4677 | General | - | CC | |  | 22.6 |  | 8.9 | 36.0 | 57.7 | 1 | | Both | | temperature, pressure, humidity, influenza epidemic and pollen count | |
| Pereira G^50^  2010 | Perth, Australia, 5 yr | 2 | 603 | Children | ICD-10J46 | CC | | 0.3 |  |  |  | 12.9 |  | 1 | | Single | | - | |
| Evans KA ^68^  2013 | New York, US,  3yr | 0 | 71 | Children | - | CC | | 0.5 |  | 8.6 | 15.4 |  | 55.9 | 0 | | Both | | Temperature, humidity | |
| Gleason JA^87^  2014 | New Jersey, US  4yr | 5 | 21,854 | Children | ICD-9 J493 | CC | |  |  | - |  |  | - | 1 | | Both | | long-term trend, seasonality, temperature, humidity, day-of-week, holiday, viral upper respiratory  infections | |
| Sacks JD^88^  2014 | North Carolina, US,  3yr | 5 | 121,621 | General | ICD-9 J493 | CC | |  |  |  |  |  | 93.4 | 1 | | Cumulative | | long-term trend, seasonality, temperature, humidity, day-of-week, holiday, viral upper respiratory  infections | |
| Raun LH^89^  2014 | Houston, Texas, US, 7yr | 3 | 11,754 | General | - | CC | | 0.3 |  | 10.7 | 4.3 | 21.6 | 76.7 | 0 | | Both | | long-term trend, seasonality, temperature, humidity, day-of-week, holiday, viral upper respiratory  infections | |
|  |  |  |  |  |  |  | |  |  |  |  |  |  |  | |  | |  | |
|  |  |  |  |  |  |  | |  |  |  |  |  |  |  | |  | |  | |
|  |  |  |  |  |  |  | |  |  |  |  |  |  |  | |  | |  | |
| **Study** | **Location**  **/Period** | **Quality score** | **Sample size** | **Population** | **Diagnosis standard** | **Type of study** | | **Pollutants and concentration** | | | | | | | **Measurement quality**  **score (0-1 point)** | | **Lag pattern(single day/ mean days)** | **Adjustments (long-term trend, seasonality, temperature, humidity, pressure, day for the week, holiday and influenza epidemics )** | |
|  |  |  |  |  |  |  | | **CO** | **PM_10_** | **PM_2.5_** | **SO_2_** | **NO_2_** | **Ozone** | |  | |  |  | |
| **Studies with hospital admission indices** | | | | | | | | | | | | | | | | | | | |
| Samoli E^7^  2010 | metropolitan area of Athens, 3yr | 5 | 3601 | Children | ICD-9: 493 | TS |  | | 43.9 |  | 16.8 | 84.8 | 70.9 | | 1 | | Single | long-term trends, seasonality, temperature, humidity, day of week, public holidays and influenza epidemics | |
| Wong TW^19^  1999 | Hong  Kong, 1yr | 5 | - | General | ICD-9 J493 | TS |  | | 45.0 |  | 17.1 | 51.4 | 24.2 | | 1 | | Both | long-term trends, seasonality, temperature, humidity, days-of-week, public holiday | |
| Fusco D^20^  2001 | Rome, Italy 2yr | 5 | 4635 | General | ICD-9 J493 | TS | 3.6 | |  |  | 9.1 | 86.7 | 27.0 | | 1 | | Single | long-term trends, seasonality, temperature, humidity, day- of-week, public holiday, influenza | |
| Morgan G^21^  1998 | Sydney, Australia  5yr | 5 | - | General | ICD-9 J493 | TS |  | | 19.2 |  |  | 30.8 | 53.6 | | 1 | | Single | long-term trends, seasonality, temperature, humidity, day-of-week, holiday | |
| Anderson HR^24^  1998 | London, UK 5yr | 5 | 63039 | General | ICD-9 J493 | TS |  | |  |  | 32 | 76.4 | 33.2 | | 1 | | Both | Long time trends, temperature, humidity, seasonality, days of the week, public holidays and influenza epidemics | |
| Lee SL^28^  2006 | Hong Kong, 5yr | 5 | 26663 | Children | ICD-9 J49 | TS |  | | 56.1 | 45.3 | 17.7 | 64.7 | 28.6 | | 1 | | Single | Long term trend, seasonality,  temperature, humidity, day-of-week, public holiday, influenza epidemic | |
| Petroeschevsky A^34^  2001 | Brisbane, Australia, 8yr | 5 | 13246 | Children + adults | ICD-9 J493 | TS |  | |  |  | 11.7 | 28.5 | 40.7 | | 1 | | Both | Long term trend, seasonality,  temperature, humidity, day-of-week, influenza epidemic | |
| Ko FWS^38^  2007 | Hong Kong, China, 6yr | 5 | 69176 | General | ICD-9 J493 | TS |  | | 52.5 | 36.4 | 18.8 | 53.2 | 43.4 | | 1 | | Both | Long term trend, seasonality,  temperature, humidity, day-of-week, public holiday | |
| Krmpotic D^39^  2011 | Zagreb, Croatia, 3yr | 5 | 808 | Adults | ICD-10 J493 | TS | 0.8 | | 39.0 |  |  | 30.3 |  | | 1 | | Single | Long term trend, seasonality,  temperature, humidity, day-of-week, influenza epidemic | |
| Romero-Placeres M^59^  2004 | Habana, Cuba, 2yr | 5 | 44 029 | General | ICD-9 J45 | TS |  | | 59.2 |  | 21.1 |  |  | | 1 | | Single | long-term trend, seasonality, temperature, humidity, day-of-week, public holiday | |
| Morgan G^71^  2010 | Sydney, Australia, 8.5yr | 5 | 65448 | Children  + adults | ICD-9 J493 | TS |  | | 62.0 |  |  |  |  | | 1 | | Single | long-term trend, seasonality, temperature, humidity, day of week, influenza epidemic | |
| Fletcher T^80^  2000 | San Paulo, Brazil, 2yr | 4/5 | - | Children | ICD-9 J493 | TS | 5.8 | | 64.9 |  | 18.3 | 174.3 | 63.4^c^ | | 1 | | Single | long-term trend, seasonality, temperature, humidity, day of week, holiday | |
| Son JY^5^  2013 | Seoul, Busan, Incheon, Daegu, Daejeon, gwangju, and Ulsan, Korea, 6yr | 4 | - | General | ICD-9 J493 | TS | 1.0^a^ | | 52.4 |  | 15.7 | 48.0 | 71.7 | | 1 | | Both | long-term trend, seasonality, temperature, humidity, pressure, day-of-week | |
| Andersen ZJ^8^  2008 | Copenhagen, Denmark, 3yr | 4 | - | Children | ICD-9: 493 | TS | 0.36 | | 24.0 | 10.0 |  | 22.6 | 51.4 | | 0 | | Cumulative | long-term trends, seasonality, temperature, humidity, day-of-week, public holidays, influenza epidemics | |
| Kim SY ^10^  2012 | Denver, US, 4yr | 4 | 10590 | General | ICD-9: 493 | TS |  | |  | 8.0 |  |  |  | | 1 | | Single | long-term trend, seasonality, temperature, humidity, day-of-week | |
| Lee JT ^11^  2002 | Seoul, Korea, 2 yr | 4 | 6436 | General | ICD-10 J45-J46 | TS | 2.3 | | 64.0 |  | 22 | 64.7 | 77.1 | | 1 | | Cumulative | long-term trend, seasonality, temperature, and humidity, day-of-week | |
| Delfino RJ ^13^  1994 | Montreal, Canada, 4yr | 3 | 10385 | General | ICD-9: 493 | TS |  | | 29.5 |  |  |  |  | | 0 | | Single | long-term trend, seasonality, temperature, humidity, day-of-week | |
| Ye F25  2001 | Tokyo, Japan, 15yr | 3 | 2200 | Elderly | ICD-9 J493 | TS |  | | 46.0 |  |  |  |  | | 1 | | Single | Long time trends, seasonality, temperature | |
| Lee JT36  2006 | Seoul, Korea, 1 yr | 4 | 2952 | Children | ICD-9 J493 | TS | 8.0^a^ | | 135.2 |  | 25.0 | 156.2 | 63.9 | | 1 | | Single | Long term trend, seasonality,  temperature, humidity, day-of-week, | |
| Lin M ^40^  2004 | Vancouver, British Columbia, Canada, 12yr | 4 | 3754 | Children | ICD-9 J493 | TS | 1.2 | |  |  | 13.6 | 38.4 | 60.0^c^ | | 1 | | Both | Long term trend, seasonality,  temperature, humidity, day-of-week | |
| Schouten JP^45^  1996 | Amsterdam, the Netherlands, 12yr | 4 | - | General | ICD-9 J493 | TS |  | |  |  | 28.0 | 50.0 | 69.0 | | 0 | | Both | long-term trend, seasonality, temperature, humidity, day-of-week, public holiday, influenza epidemics | |
| Silverman RA ^52^  2010 | New York, US, 7yr | 4 | 75383 | General | ICD-9 J493 | TS |  | |  | 13．0 |  |  | 87.9 | | 1 | | Cumulative | long-term trend, seasonality, temperature, humidity, day-of- week | |
| Amancio CT^65^  2012 | San Paulo, Brazil, 2yr | 4 | 841 | Children | ICD10: J45 | TS |  | | 25.2 |  | 4.6 |  | 74.3 | | 1 | | Single | long-term trend, seasonality, temperature, humidity, day-of-week | |
| Sheppard L ^70^  1999 | Seattle, Washington, 8yr | 3/4 ^b^ | 7837 | Children  + adults | ICD-9 J493 | TS | 2.3 | | 31.5 | 16.7 | 22.9 |  | 65.1 | | 1/0^a^ | | Single | long-term trend, seasonality, temperature, humidity, day-of-week | |
| Neidell M ^75^  2010 | Southern California, 8yr | 4 | - | General | ICD-9 J493 | TS |  | |  |  |  |  | 175.7 | | 1 | | Cumulative | long-term trend, seasonality, temperature, humidity, day-of-week | |
| Fung KY^81^  2005 | London, Ontario, Canada, 5yr | 4 | ? | Children +  adults | ICD-9 J493 | TS |  | | 38.0 |  |  |  |  | | 1 | | Both | long-term trend, seasonality, temperature, humidity, day-of-week | |
| Hua J^83^  2014 | Shanghai, China,  7yr | 4 |  | Children | ICD10: J45 | TS |  | |  | 34.0 |  |  |  | | 1 | | cumulative | long-term trend, seasonality, temperature, humidity | |
| Walters S ^9^  1994 | Birmingham, UK, 2yr | 2 | - | General | ICD-9: 493 | TS |  | |  |  | 39.1 |  |  | | 1 | | Cumulative | Non declared | |
| Magas OK ^35^  2007 | Oklahoma city metropolitan area, US, 3yr | 1 | 1270 | Children | ICD-9 J493 | TS |  | |  |  |  | - |  | | 0 | | Single | Temperature, day-of-week, humidity, holiday | |
| Barnett AG ^53^  2005 | Australia (Brisbane, Canberra,  Melbourne, Perth, and Sydney), New  Zealand (Auckland, Christchurch), 3yr | 5 | - | Children | ICD-9 J493 | CC | 1.1 | | 17.6 | 9.4 | 7.0 | 17.6 | 49.6 | | 1 | | Cumulative | long-term trend, seasonality, temperature, humidity, day for the week, holiday and influenza epidemics | |
| Yang CY^17^  2007 | Taipei, 8yr | 4 | 25602 | General | ICD-9 J493 | CC | 1. 7 | | 49.0 |  | 12.3 | 63.0 | 44.0 | | 1 | | Cumulative | Long-term trend, seasonality, temperature, humidity | |
| Tsai SS^19^  2006 | Kaohsiung, 8yr | 4 | 17682 | General | ICD-9 J493 | CC | 1.00 | | 76.7 |  | 27.1 | 55.9 | 56.3 | | 1 | | Cumulative | long-term trends, seasonality, temperature, humidity | |
| Lin M ^37^  2003 | Toronto, Ontario, Canada. 13yr | 4 | 7319 | Children | ICD-9 J493 | CC | 1.5 | |  |  | 15.3 | 51.8 | 65.1^c^ | | 1 | | Both | Long term trend, seasonality,  temperature, humidity, day-of-week, | |
| Lin M ^44^  2002 | Toronto, Canada, 14yr | 4 | 7319 | Children | ICD-9 J493 | CC |  | | 30.2 | 18.0 |  |  |  | | 1 | | Both | Long-term trend, seasonality, temperature, humidity, day-of-week | |
| Iskandar A^68^  2012 | Copenhagen, Denmark, 8yr | 4 | 8226 | Children | ICD-10 J45-46 | CC |  | | 26.2 | 10.3 |  | 23.3 |  | | 1 | | Cumulative | long-term trend, seasonality, temperature, humidity, day-of-week | |
| Cheng MH^85^  2014 | Taipei, China,  5yr | 3 | 10,440 | General | ICD-9 J493 | CC |  | |  | 30.0 |  |  |  | | 1 | | Cumulative | long-term trend, seasonality, temperature, humidity | |
| Cai J^86^  2014 | Shanghai, China  7yr | 4 | 15,678 | General | ICD-10 J45 | TS |  | | 88.0 |  | 45.0 | 60.0 |  | | 1 | | Both | long-term trend, seasonality, temperature, humidity, day-of-week | |

| **Study** | **Location/Period** | **Quality score** | **Sample size** | **Population** | **Diagnosis standard** | **Type of study** | **Pollutants and concentration** | | | | | | **Measurement quality**  **score (0-1 point)** | **Lag pattern(single day/ mean days)** | **Adjustments (long-term trend, seasonality, temperature, humidity, pressure, day for the week, holiday and influenza epidemics )** |
| --- | --- | --- | --- | --- | --- | --- | --- | --- | --- | --- | --- | --- | --- | --- | --- |
|  |  |  |  |  |  |  | **CO** | **PM_10_** | **PM_2.5_** | **SO_2_** | **NO_2_** | **Ozone** |  |  |  |
| **Studies with both emergency room visit and hospital admission indices** | | | | | | | | | | | | | | | |
| Chew FT ^33^  1999 | Singapore, 5yr | 4 | 6000(HA), 23000(ER) | Children | ICD-9 J493 | TS |  |  |  | 38.1 | 18.9 |  | 1 | Single | long-term trend, seasonality, temperature, humidity, day-of-week |
| Sluaghter JC^62^  2005 | Spokane, Washington DC, US, 7yr | 4 | 2191/2373 | General | ICD-9 J493 | TS | 1.6-3.8 | 7.9-41.9 | 4.2-20.2 |  |  |  | 1 | Single | long-term trend, seasonality, temperature, humidity, day-of-week |
| Smargiassi A ^26^  2009 | 0.5–7.5 km of the refinery stacks, Montreal Canada, 8yr | 4 | 1579/263 | Children | ICD-9 J493 | CC |  |  |  | 12.3 |  |  | 1 | Both | Long time trends, seasonality, temperature,  humidity, day-of-week |
| Li S ^63^  2001 | Detroit, Michigan, 2yr | 4 | 7063 | Children | ICD-9 J493 | CC | 0.5 |  | 15.0 | 10.8 | 34.4^c^ |  | 1 | Both | long-term trend, seasonality, temperature, humidity, day-of-week |

a: 8-hr maximum concentration; c: daily averaged concentration; ICD: International Classification of Diseases; ICPC2: International Classification of Primary Care 2; GP: GP’s house calls; NAEPP: National Asthma Education and Prevention Program; PC: primary care visits; TS: time-series study; CC: case cross over studies.

The default concentration for CO, NO_2_, SO_2_, PM_10_ and PM_2.5_ is 24-h averaged concentration, while 8-hr maximum concentration for ozone
